# Effectiveness of Multi-Criteria Optimization-based Trade-Off exploration in combination with RapidPlan for head & neck radiotherapy planning

**DOI:** 10.1186/s13014-018-1175-y

**Published:** 2018-11-23

**Authors:** Eliane Miguel-Chumacero, Garry Currie, Abigail Johnston, Suzanne Currie

**Affiliations:** Beatson West of Scotland Cancer Centre, Radiotherapy Physics, NHS Greater Glasgow and Clyde, 1053 Great Western Road, Glasgow, G12 0YN UK

**Keywords:** Multi-criteria optimisation, Trade-offs exploration, Head and neck cancer, Knowledge-based treatment planning, RapidPlan, Volumetric-modulated arc therapy

## Abstract

**Background:**

A new strategy is introduced combining the use of Multi-Criteria Optimization-based Trade-Off Exploration (TO) and RapidPlan™ (RP) for the selection of optimisation parameters that improve the trade-off between sparing of organs at risk (OAR) and target coverage for head and neck radiotherapy planning. Using both approaches simultaneously; three different workflows were proposed for the optimisation process of volumetric-modulated arc therapy (VMAT) plans. The generated plans were compared to the clinical plans and the plans that resulted using RP and TO individually.

**Methods:**

Twenty clinical VMAT plans previously administered were selected. Five additional plans were created for each patient: a clinical plan further optimised with TO (Clin+TO); two plans generated by in-house built RP models, RP_1 with the model built with VMAT clinical plans and RP_TO with the model built with VMAT plans optimised by TO. Finally, these last two plans were additionally optimised with TO for the creation of the plans RP_1 + TO and RP_TO^+^ respectively. The TO management was standardised to maximise the sparing of the parotid glands without compromising a clinically acceptable PTV coverage. Resulting plans were inter-compared based on dose-volume parameters for OAR and PTVs, target homogeneity, conformity, and plans complexity and deliverability.

**Results:**

The plans optimised using TO in combination with RP showed significantly improved OAR sparing while maintaining comparable target dose coverage to the clinical plans. The largest OAR sparing compared to the clinical plans was achieved by the RP_TO^+^ plans, which reported a mean parotid dose average of 15.0 ± 4.6 Gy vs 22.9 ± 5.5 Gy (left) and 17.1 ± 5.0 Gy vs 24.8 ± 5.8 Gy (right). However, at the same time, RP_TO^+^ showed a slight dose reduction for the 99% volume of the nodal PTV and an increase for the 95% (when comparing to the clinical plans 76.0 ± 1.2 vs 77.4 ± 0.6 and 80.9 ± 0.9 vs 79.7 ± 0.4) but remained within clinical acceptance. Plans optimised with RP and TO combined, showed an increase in complexity but were proven to be deliverable.

**Conclusion:**

The use of TO combined with RP during the optimisation of VMAT plans enhanced plan quality the most. For the RP_TO^+^ plans, acceptance of a slight deterioration in nodal PTV allowed the largest reduction in OAR dose to be achieved.

## Background

The challenge in radiotherapy planning is to manage the compromise between tumour irradiation and sparing of healthy tissue. The planning objectives involved can be inherently contradictory, which restricts an optimal result for all objectives at the same time. This situation is reflected in the irradiation of head and neck cancer (HNC) where there are many organs at risk (OAR) and their close proximity to the target volume makes it difficult to achieve clinically acceptable target coverage without radiation-induced toxicity and decreased of quality of life [1–3].

For the treatment of HNC, volumetric-modulated arc therapy (VMAT) has shown good dose conformity and sparing of OAR with a decrease in treatment delivery time compared to other techniques [4–7]. For this inverse planning technique, the desired dose is prescribed for the planning target volume (PTV) and for the surrounding structures the tolerated dose is defined; then through an iterative optimisation process a plan is computed. The optimisation result is the plan which best achieves the stated objectives by minimising a cost function associated with them. However, this result is often not satisfactory since the compromise between the planning goals might not be clinically acceptable and the optimisation objectives may not adequately describe the clinical situation. Different objective settings are tried until an acceptable solution is found, which is time consuming and may result in sub-optimal treatment plans. For the solution of this inverse problem, a Multi-Criteria Optimisation (MCO) approach has been proposed as a more efficient method [8–15]. Whereas single objective function optimisation problems have one best solution, in MCO a vector of objective functions is optimised resulting in many best-compromised solutions that describe a mathematical construct, an approximation of the Pareto surface. In practice this is composed of alternative plans that are created, where each of them will prioritise a specific optimisation objective over all others. Therefore, each alternative plan is optimal in that one objective can only be improved by deteriorating others [16].

Eclipse treatment planning system (TPS) Varian Medical Systems, Palo Alto, CA, version 15.5 introduces the tools for a MCO approach based on trade-offs exploration (TO). A copy of an initial plan optimisation, called the balanced plan, is used together with the chosen N optimisation objectives as the base for the subsequent generation of the 3 N + 1 alternative plans for objective. The user can then explore the trade-offs along the possible solutions and select the plan that best fulfils the treatment goals. The initial plan influences the subsequent Pareto frontier approximation, therefore, trade-offs exploration around a starting promising plan is desirable [16, 17].

Recently, knowledge-based radiotherapy treatment planning has been incorporated into clinical practice. RapidPlan™ (RP) (Eclipse v15.5) operates with a knowledge-based treatment planning algorithm that is able to generate dose volume histogram (DVH) estimates for a new patient geometry by analysis of a database of previous plans. The DVH estimates are then translated into optimisation objectives parameters for intensity modulated radiotherapy (IMRT) or VMAT plans. The resulting plans have shown to reduce planner interaction while improving the quality and consistency [18, 19]. However, the quality of the plans generated for new patients depends highly on the quality and robustness of the plans in the training cohort [20].

In this study the key question addressed was how to maximise the performance of RP and TO for the radiotherapy treatment of HNC. We suggested a combination of both approaches, RP and TO used simultaneously during the optimisation process of a plan. Three different workflows were proposed for the VMAT planning of HNC patients and an inter-comparison of the resulting plans was performed. At the same time, these were compared with the plans optimised using RP and TO on their own and the clinical plans for reference.

## Methods

### Patient data

Clinical HNC plans previously administered were available from a database with tumours originating in various sites and stages, i.e. tongue, glottis, supraglottis, lip, oral cavity, nasal cavity, tonsillar region, larynx, pharynx (oro, hypo and naso), neck and parotids. Plans for the region of optical structures were not included. The clinical plans were generated with the in-house optimisation template (based on the plan constraints shown on Table 1) and expert planners modified the optimisation parameters as necessary. The planned target volumes were: the main tumour (PTV1), and neck nodes encompassed by the low-risk planning treatment volume (PTVLR). Dose levels of 65Gy and 54Gy in 30 fractions were respectively prescribed. Both PTVs were created with 3–5 mm margins from the Clinical Target Volume (CTV). Planning organ at risk volumes (PRVs) were used for spinal cord and brainstem by expanding from these structures. The planning technique was the Varian VMAT solution RapidArc™, utilising 2 full coplanar arcs with the collimator rotated to 30 and 330 degrees, and 6MV photons. Plan calculations were made using the Eclipse™ Photon Optimiser and Anisotropic Analytic Algorithm version 15.5.07. Aperture Shape Control was set to “Moderate” and all had the same Normal Tissue Objectives (NTO). The target volumes and OAR constraints followed in this study for all the sets of plans are shown on Table 1 and are based on the Phase 3 PARSPORT trial specifications [21, 22].

**Table 1:** Dose-volume constraints for the PTVs and OAR

PTV/Organ	Dose-Volume constraint
PTV1 (65Gy prescribed in 30 fractions)	99% volume more than 90% of the dose 95% volume more than 95% of the dose 5% volume less than 105% of the dose 2% volume less than 107% of the dose
PTVLR (54Gy prescribed in 30 fractions)	99% volume more than 90% of the dose 95% volume more than 95% of the dose
PRV Spinal cord	The dose for 1% of the volume is limited to 44Gy and no part should receive more than 48 Gy
PRV Brainstem	48 Gy was restricted to no more than 1% of the volume
Optic Nerve	50 Gy was restricted to no more than 1% of the volume
Optic Chiasm	50 Gy was restricted to no more than 1% of the volume
Parotids	The maximum mean dose is limited to 24 Gy
Larynx	The maximum mean dose is limited to 40 Gy

Two different sets of plans were used in this work. Set_1 consisted of a collection of 54 plans that were selected randomly from the database for the creation of the RP models described in the methods section part III. These plans had a mean PTV1 volume of 325.0 cc (range 68.6–786.6 cc) and mean PTVLR volume of 182.0 cc (range 0–375.7 cc).

Set_2 included 20 different patients; these had a mean PTV1 volume of 291.8 cc (range 75.5–1069.4 cc) and a mean PTVLR volume of 274.2 cc (range 124.7–389.0 cc). Set_2 was selected from cases with no full parotid involvement (2 Tonsil, 6 Larynx, 5 Pharynx, 5 oral, and 2 face), with mean volume overlap of parotids and PTVs of 19.9%, (range 0–40.8%). Set 2 was used for the creation, comparison, and analysis of plans using TO and/or RP.

### Trade-offs exploration optimisation

The inclusion of MCO in radiotherapy planning aims to allow the exploration of the trade-offs of the treatment objectives in an efficient way to then select a plan that best fulfils the prescribed clinical goals. To commence, it is required to have a starting plan (that will be the centre of the approximation of the Pareto surface). The optimisation objectives used in inverse radiotherapy planning are based on dose statistics to regions of interest, for instance:

- Upper or lower objectives: dose-volume points that define the maximum or minimum dose that a structure may receive.
- Mean objective: mean dose level that should not be exceeded for a structure.
- Line objective: it is located under the lower bound of the DVH estimate range. If the OAR overlaps with the target, the highest upper objective of the target is the defining level in that region.

A starting plan will be used together with a selection of the optimisation objectives for trade-off exploration as the base for a generation of alternative plans.

The optimisation objectives selected for the trade-off exploration of this study were: right and left parotids line dose or mean dose (for the Clinical plans), PRV brainstem upper point (D_max_), PRV spinal cord combined (upper points D_1_ and D_max_), and larynx line dose only when the tumour did not originate in it. Upper and lower point objectives were selected for PTV1 and lower point objectives for PTVLR [23].

After the calculation of the alternative plans, a slider for each selected objective is displayed. The sliders represent the range for the objective covered by the balanced and alternative plans. A selector in the slider can be manipulated to explore the trade-offs, when it is moved to the left the associated objective improves. The manipulation of one slider automatically affects the position of other selectors depending on the trade-offs. If the planner wants to restrict the range of a specific objective a component in the slider called restrictor is used, this keeps the selector within the new range influencing how the cost is distributed during the trade-off exploration; otherwise, the algorithm aims to distribute the cost of the improvement evenly among other criteria [16]. The feedback of the exploration was constantly monitored through visual assessment of the DVHs, an axial view of the dose distribution on patient anatomy, and in the clinical protocol attached to the plan (see Fig. 5 in Appendix). Once satisfied with the result, the DVHs and the spatial distribution of the dose that resulted from the trade-offs were used as optimisation objectives parameters for the creation of a deliverable VMAT plan and the final dose was calculated.

We prioritised a dosimetric reduction on the parotid glands [24–26]. Therefore, on the alternative plans that were generated, the management of the trade-offs was standardised as follows:

1. A restrictor to stop further dose increase to spinal cord and brainstem was applied; this action still allowed their initial dose to reduce as a consequence of other structures improvement.
2. The mean dose to right and left parotids was reduced. The limit for this was meeting the prescribed dose constraints for the PTVs.
3. Mean dose to larynx was reduced with the least importance over the other organs and only when its effect would not compromise the target constraints.

To begin the study, Set_2 plans were selected as the starting plans and optimised with TO to create the Clin+TO plans following the procedure described above.

### RapidPlan with trade-offs optimisation

For RapidPlan knowledge-based planning a model needs to be created. The model configuration comprises two phases: data extraction and model training. During the first phase, the data related to the structure set, dose, and field geometry of each plan included is extracted; this information is used to train the model which will then estimate DVHs for a new plan based on the information obtained from the data set of plans [27].

Once the model is trained, it will be used together with the structure set of the new plan and the dose specifications for the target to provide a DVH estimation range for each of the OAR; also it will provide the optimisation objectives parameters needed to try to achieve the estimation.

Two RP models for HNC were generated using the dosimetric and geometric data from Set_1. The first model (RP_1) was trained with the 54 standard clinical VMAT plans. The second model (RP_TO) used the same VMAT plans but this time further optimisation was carried out using TO (following the process in Section II) prior to adding them to the model. Set_1 consisted of plans previously used for the head and neck RP model that is currently in use at our clinic, therefore, these plans are clinically acceptable and the model was processed for elimination of outliers. Both models were generated by training the two PTV targets and 12 OAR structures (PRV brainstem, larynx, lenses, optic nerves, orbits, parotids, PRV spinal cord, and trachea). The optimisation objectives selected are shown in Table 5 in Appendix.

**Fig. 1:**
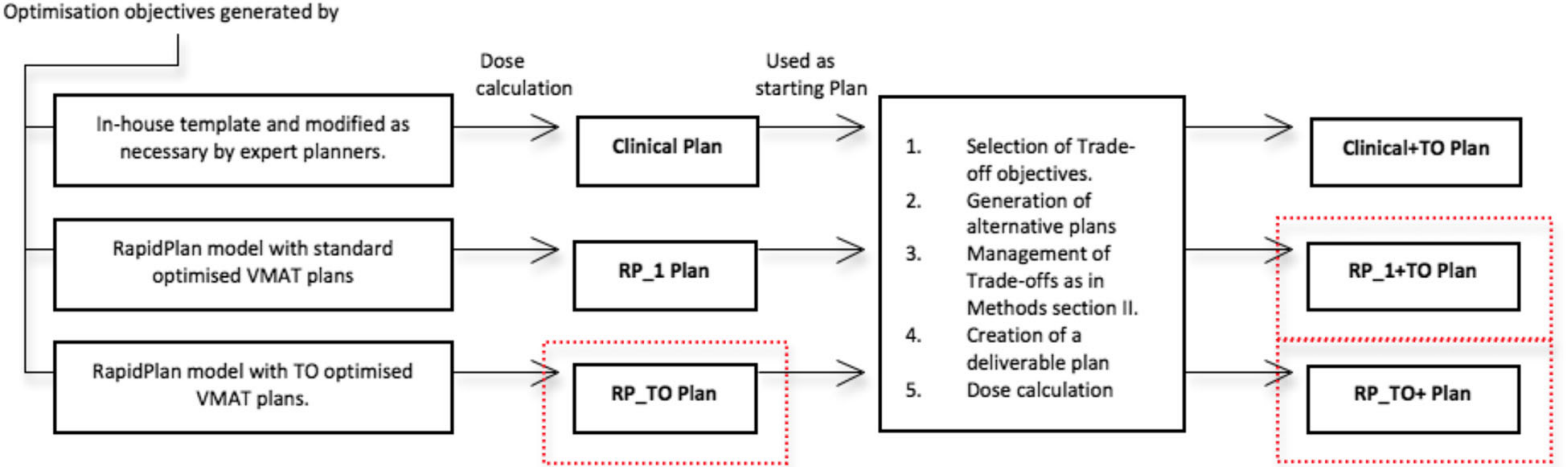
Planning workflow of: Clinical, Clinical+TO, RP_1, RP_1 + TO, RP_TO, and RP_TO^+^. (See in red proposed workflows)

The two models were used to generate a plan for each patient of Set 2 without manual intervention*,* RP_1 and RP_TO. The resulting plans were then used as the initial plans for a further optimisation using TO for the creation of the last two types of plans for each patient, RP_1 + TO and RP_ TO^+^ (following the same trade-offs exploration procedure described on section II). In this way, the trained models suggested the optimisation objectives parameters for an initial plan that would be the centre of the later approximation of the Pareto surface. Figure 1 summarizes the workflows used for the generation of the plans created for this study. All plans from this part and section II were normalized 100% to target mean, and the dosimetric data was collected.

### Data comparison and analysis

For the clinical plans and the 5 different plans generated for each patient, PTVs and OAR doses were evaluated following the dose-volume constraints specified. Additionally, the homogeneity of the PTVs was assessed by calculating the Homogeneity Index according to Eq. 1 [28].

1 $$ HI=\left(\frac{D_{5\%}}{D_{95\%}}\right) $$

Also, the dose delivered to normal tissue was assessed by Eq. 2 [29].

2 $$ \mathrm{Conformity}\kern0.5em \mathrm{Index}\kern0.5em (CI)=\frac{Vx\%}{VPTVs} $$

Where Vx% is the volume receiving at least x% of the dose and VPTVs is the sum of both target volumes PTV1 and PTVLR. This was calculated for V100%, V80%, V50%, and V10%.

To investigate plan deliverability, RadCalc version 6.3 was used to quantify several complexity parameters: the total number of monitor units (MU), the modulation factor (MF) and the average leaf pair opening (ALPO) [30, 31].

For the plans that resulted in lowest MF, plan specific quality assurance (QA) measurements were performed at the treatment room for further evaluation of the delivered dose. A Varian TrueBeam Slim linear accelerator was used and the phantom Octavius 729 detector array together with the Octavius 4D rotating unit. A comparison between the delivered and the planned dose was performed with a 3D gamma evaluation (global and local) using the verification software VeriSoft 6.1 (6.1.0.46) and an acceptance of 95% points passing the criteria of 3 mm for the distance to agreement (DTA) and a dose difference tolerance level of 3% [32, 33]. For all above comparative analysis, the threshold for inclusion was set at 10% of the maximum dose.

Significant differences between the 6 final sets of plans were assessed for all the investigated parameters by the Wilcoxon signed-rank test at a level of 0.05.

## Results

### Dosimetric evaluation

All plans resulting from implementing TO and/or RP were deemed clinically acceptable according to the criteria of PTV coverage and OAR doses (Table 1). While maintaining comparable target coverage, the sparing of the OAR improved. Table 2 lists the dosimetric parameter values (average ± standard deviation) obtained for the PTVs and OAR for the HNC plan sets following each of the 5 planning procedures described, and for the clinical plans; the letters indicate significant difference at a level of 0.05 when inter-comparing these techniques. The HI, CI, and the number of parameters that failed to meet the constraints for each procedure, and the number of plans that correspond to these failures, are also displayed (for further detail see Table 6 in Appendix). Additionally, doses were investigated for larynx and trachea and are reported in Table 7 in Appendix.

**Table 2:** Summary of average ± s.d. doses to PTVs and OAR for the 20 patients in Set_2

Structure	Parameter	a - Clinical	b - Clinical + TO	c - RP_1	d - RP_1 + TO	e - RP_TO	f - RP_ TO^+^
PTV 1	D_99%_ > 90% *p* < 0.05	93.7 ± 0.9 c	94.1 ± 2.0	94.2 ± 1.2 a,e	93.8 ± 2.5	93.6 ± 1.5 c	93.7 ± 2.3
	D_95%_ > 95% *p* < 0.05	96.1 ± 0.6 b,c,d	96.8 ± 0.8 a,c,e,f	96.3 ± 0.7 a,b,e	96.6 ± 1.0 a,e	96.0 ± 0.9 b,c,d,f	96.4 ± 0.9 b,e
	D_5%_ < 105% *p* < 0.05	102.9 ± 0.5 b,d	102.4 ± 0.5 a,c,e,f	102.7 ± 0.4 b,d,e	102.4 ± 0.7 a,c,e,f	103.1 ± 0.6 b,c,d,	102.8 ± 0.8 b,d,
	D_2%_ < 107% *p* < 0.05	103.5 ± 0.7 b,d,e	103.0 ± 0.8 a,c,e,f	103.4 ± 0.6 b,e	103.5 ± 0.9 a,e,f	103.8 ± 0.8 a,b,c,d	103.7 ± 1.2 b,d
	HI *p* < 0.05	1.07 ± 0.01 b,c,d	1.06 ± 0.01 a,c,e,f	1.07 ± 0.01 a,b,d,e	1.06 ± 0.02 a,e,f	1.07 ± 0.01 b,c,d,f	1.07 ± 0.02 b,d,e
PTV LR	D_99%_ > 74.8% *p* < 0.05	77.4 ± 0.6 c,f	77.1 ± 1.0 c,f	78.4 ± 0.7 a,b,d,e,f	77.0 ± 1.4 c,f	77.5 ± 1.0 c,f	76.0 ± 1.2 a,b,c,d,e
	D_95%_ > 78.9% *p* < 0.05	79.7 ± 0.4 b,c,d,f	81.1 ± 0.9 a,c,e	80.2 ± 0.5 a,b,d,e,f	81.2 ± 0.6 a,c,e	79.9 ± 0.6 b,c,d,f	80.8 ± 0.9 a,c,e
	HI *p* < 0.05	1.13 ± 0.02 b,c,d,f	1.16 ± 0.02 a,c,e,f	1.12 ± 0.01 a,b,d,e,f	1.16 ± 0.02 a,c,e,f	1.13 ± 0.01 b,c,d,f	1.17 ± 0.02 a,b,c,d,e
PRV Brainstem	D_Max_ < 48Gy *p* < 0.05	26.9 ± 15.7 b,c,d,e,f	24.3 ± 14.5 a,f	25.9 ± 14.8 a,d,e,f	23.2 ± 13.3 a, c,	23.9 ± 13.5 a,c,f	20.9 ± 11.6 a,b,c,e
PRV Spinal Cord	D_1%_ < 44Gy *p* < 0.05	37.6 ± 2.7 b,c,d,e,f	32.7 ± 2.9 a,c,d,f	36.4 ± 2.2 a,b,d,e,f	30.5 ± 3.1 a,b,c,f	31.5 ± 3.0 a,c,f	27.5 ± 4.7 a,b,c,d,e
	D_Max_ < 48Gy *p* < 0.05	40.4 ± 2.9 b,d,e,f	35.1 ± 2.9 a,c,f	39.3 ± 2.2 b,d,e,f	33.6 ± 3.4 a,c,f	34.3 ± 3.5 a,c,f	30.8 ± 4.9 a,b,c,d,e
Left Parotid	D_Mean_ < 24Gy *p* < 0.05	22.9 ± 5.5 b,d,e,f	16.7 ± 4.8 a,c,f	22.3 ± 5.5 b,d,e,f	16.7 ± 4.5 a,c,f	16.8 ± 4.9 a,c,f	15.0 ± 4.6 a,b,c,d,e
Right Parotid	D_Mean_ < 24Gy *p* < 0.05	24.8 ± 5.8 b,d,e,f	19.1 ± 4.4 a,c,f	24.2 ± 5.4 b,d,e,f	18.4 ± 4.7 a,c,f	19.0 ± 5.9 a,c,f	17.1 ± 5.0 a,b,c,d,e
CI	V100%/VPTVs *p* < 0.05	0.27 ± 0.08 b,d,	0.29 ± 0.09 a,c,e	0.27 ± 0.08 b,d,f	0.29 ± 0.09 a,c	0.28 ± 0.09 b,	0.29 ± 0.09 c,
	V80%/VPTVs *p* < 0.05	1.26 ± 0.14 b,d,f	1.47 ± 0.15 a,c,d,e,f	1.23 ± 0.06 b,d,f	1.40 ± 0.08 a,b,c,e	1.24 ± 0.06 b,d,f	1.41 ± 0.07 a,b,c,e
	V50%/VPTVs *p* < 0.05	2.55 ± 0.33 b,d,f	2.94 ± 0.41 a,c,d,e,f	2.49 ± 0.19 b,d,e,f	2.79 ± 0.25 a,b,c,e	2.52 ± 0.21 b,c,d,f	2.84 ± 0.29 a,b,c,e
	V10%/VPTVs *p* < 0.05	9.31 ± 1.89 b,d,f	9.55 ± 1.90 a,c,d,e,f	9.28 ± 1.84 b,d,f	9.41 ± 1.81 a,b,c,e	9.28 ± 1.81 b,d,f	9.39 ± 1.78 a,b,c,e
Right Parotid	D_Mean_ < 24Gy *p* < 0.05	24.8 ± 5.8 b,d,e,f	19.1 ± 4.4 a,c,f	24.2 ± 5.4 b,d,e,f	18.4 ± 4.7 a,c,f	19.0 ± 5.9 a,c,f	17.1 ± 5.0 a,b,c,d,e
Total Fails	Parameters	22	2	20	4	11	3
	Plans	13	2	13	3	7	2

Comparisons are based on the Wilcoxon signed-rank test and significant differences (*p*-value < 0.05) between planning methods are indicated with the letters. The last two rows show the number of plans in each group that failed to meet all the constraints and the number of parameters that were violated within them

For the plans created, it is shown in Table 2 that PTV1 reported no statistically significant dose reduction compared to the clinical plans for its 99 and 95% volumes (D_99_, D_95_) and the homogeneity index remained comparable across techniques as well. For PTVLR, the dose homogeneity was shown to be slightly affected and D_95_ increased significantly but a D_99_ significant reduction resulted only for RP_TO^+^.

**Fig. 2:**
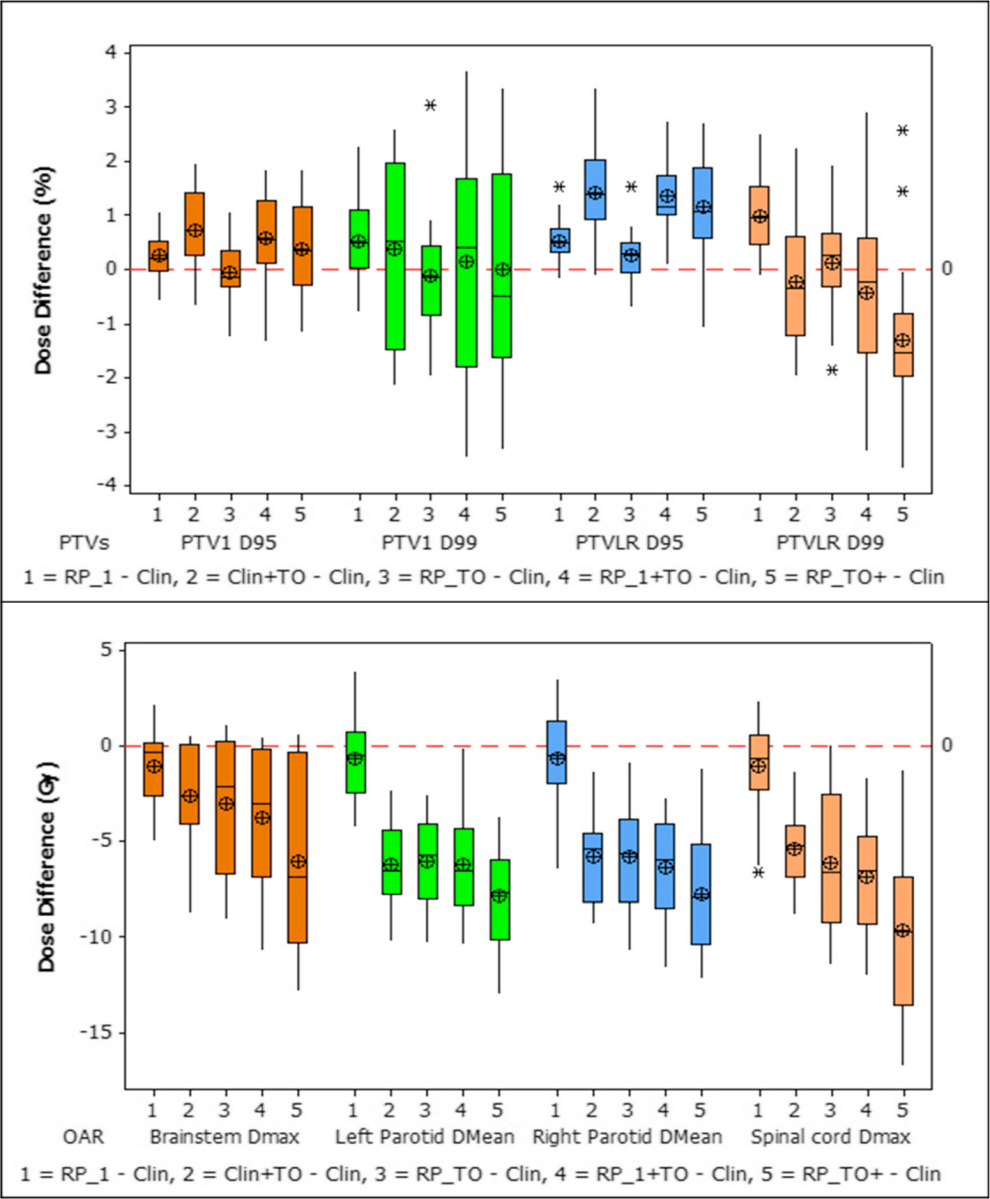
Box plot of the difference of dosimetric parameters (PTVs-above and OAR-below) between clinical and generated plans. The mean value is indicated by the ⊕ symbol, the median value by the central horizontal line, the interquartile range is represented by the box, and the outliers are indicated by the asterisks

**Fig. 3:**
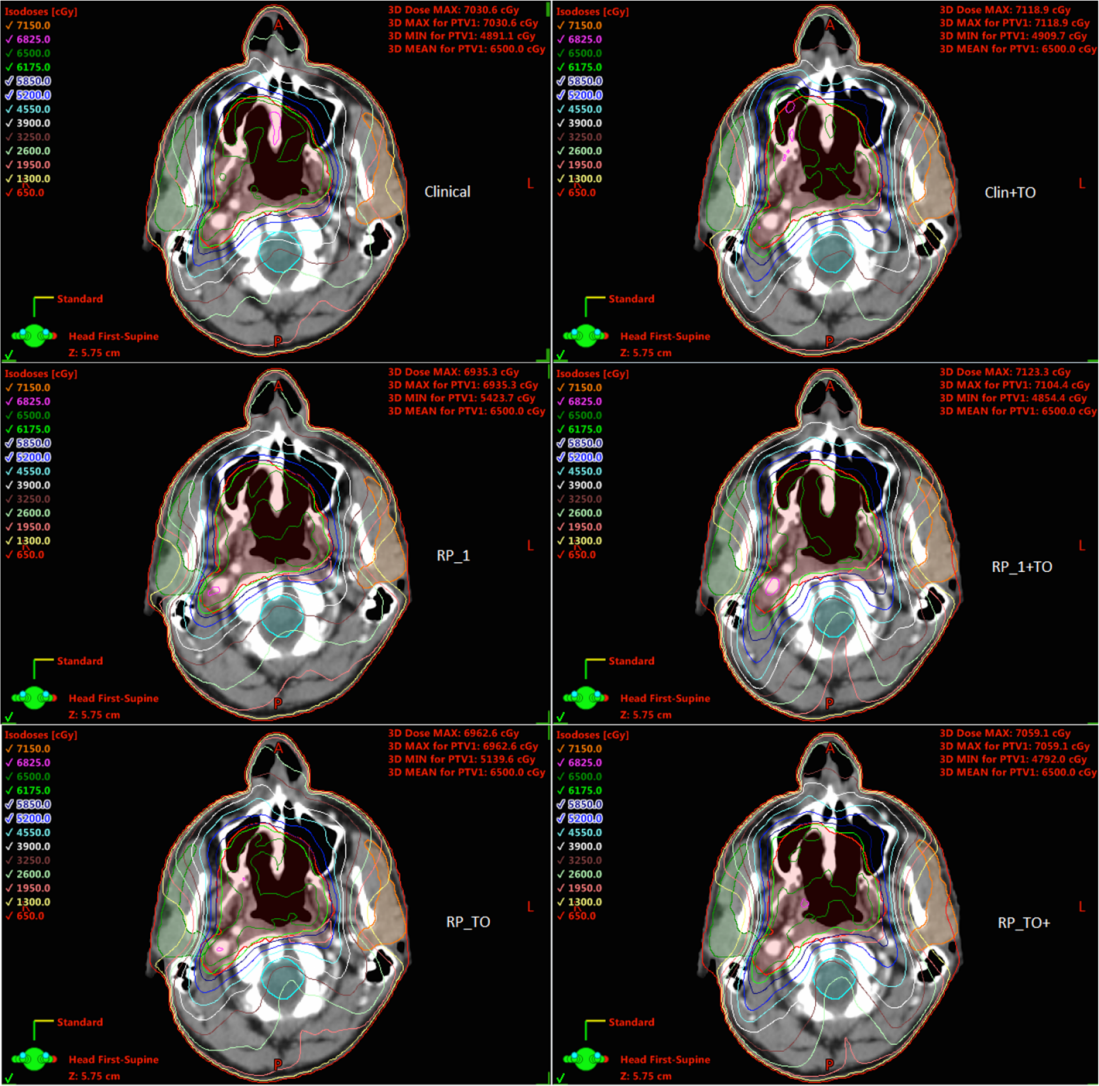
Dose distribution at the parotids level for a representative case resulting from each planning optimisation procedure

**Fig. 4:**
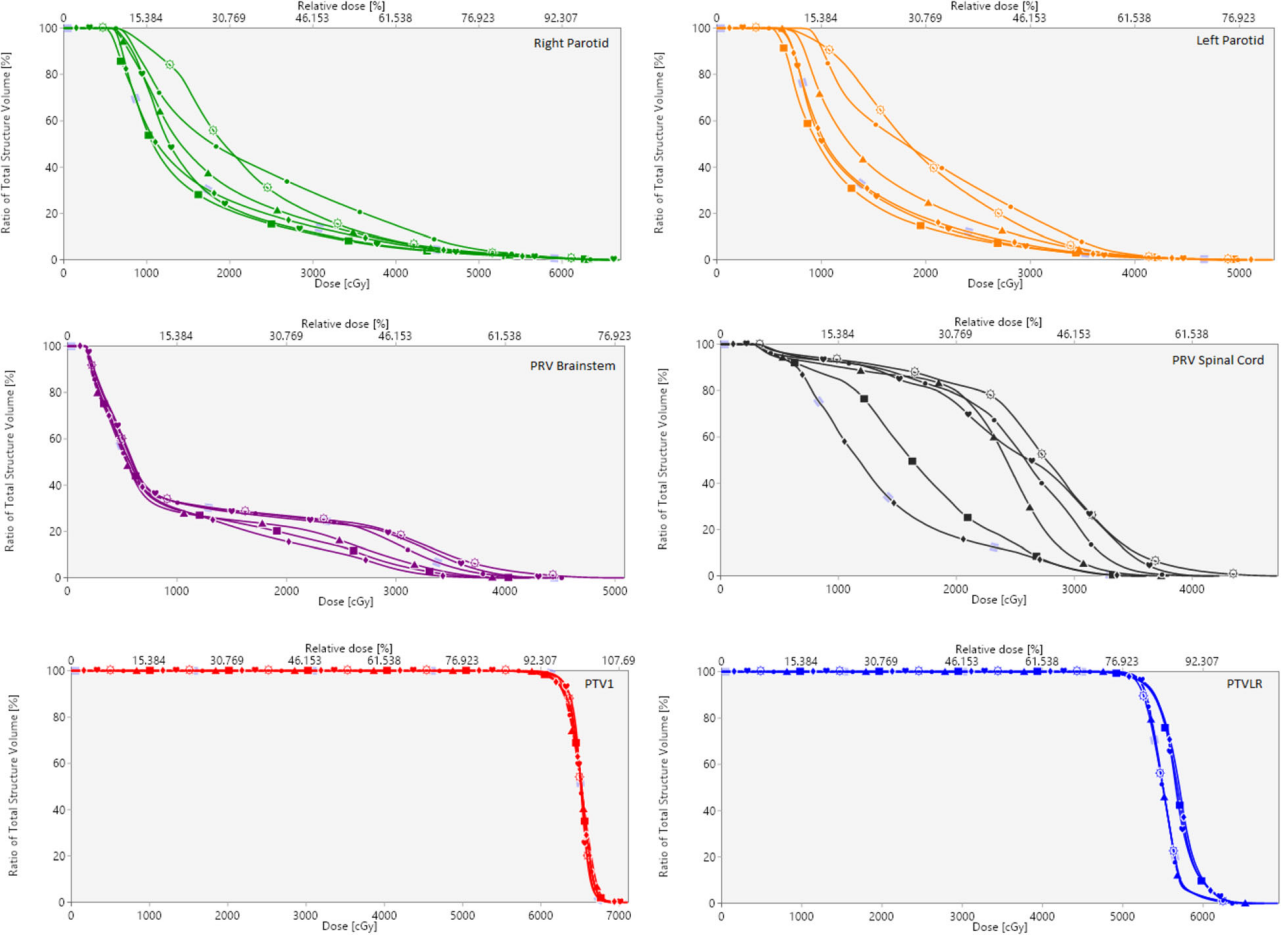
DVHs corresponding to representative case shown in Fig. 3. Clinical (suns), Clinical+TO (hearts), RP_1 (circles), RP_1 + TO (rhombus), RP_TO (triangles), RP_TO^+^ (squares)

**Fig. 5:**
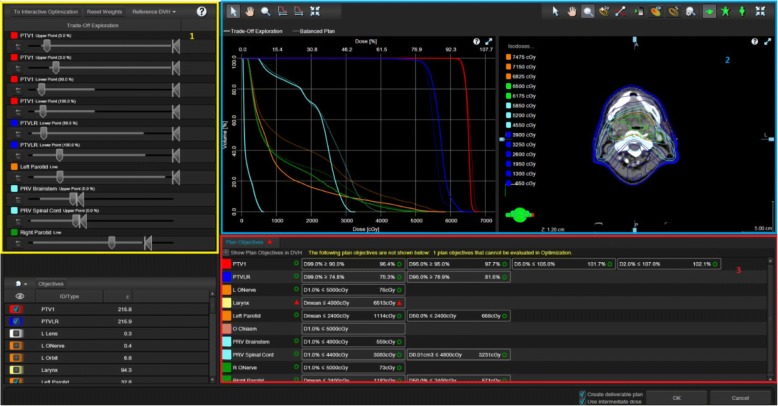
Eclipse real time plan navigation screen view during TO for one of the head and neck plans. 1-Yellow box: Trade-Offs exploration with slider bars for each selected objective. 2-Blue box: Graphical feedback of the modifications instantly monitored in the DVHs and the dose distribution view displayed. 3-Red box: Plan objectives displaying the protocol constraints for dose-volume monitoring

Figure 2 presents the dose differences obtained between the clinical plan and the other five plans created for each of the 20 patients. It is shown that the largest OAR reductions were achieved when RP and TO were implemented together, with the largest sparing for brainstem, parotid glands, and spinal cord being achieved by the RP_TO^+^ plans. For OAR, RP_1 + TO and RP_TO reported no significant difference between them. Furthermore, the combined use of RP and TO showed improvements in CI when comparing with the solely use of TO (Clin+TO plans) however whereas RP_TO plans are comparable with the clinical plans, for RP_1 + TO and RP_TO^+^ the results showed an increase. The dose distribution of a representative case is illustrated in Fig. 3, which displays all plans at the parotid level. Figure 4 shows the DVHs corresponding to the patient plans shown in Fig. 3.

### Complexity parameters evaluation

Table 3 displays the average values (and range) of the complexity parameters assessed for each group of plans. The clinical, RP_1 and Clin+TO plans, when compared, showed no significant differences in the number of MU or the MF. However, the use of TO and RP together in the three remaining types of plans led to an increase in MU and a decrease in the MF, indicating a higher complexity of the plan. As a result of this observation the two plans with the lowest MF from each of these plan categories (RP_1 + TO, RP_TO, and RP_ TO^+^) were selected for plan specific QA. Table 4 presents the analysis of the plan QA including the percentage of points passing the gamma criteria for each of them and also for their corresponding clinical plan for reference. All the plans subject to QA were reported deliverable according to the acceptance criteria given above.

**Table 3:** Complexity parameters for each group of plans (average and range)

	a - Clinical	b - Clinical + TO	c - RP_1	d - RP_1 + TO	e - RP_TO	f - RP_ TO^+^
ALPO (cm) *p* < 0.05	3.37 (2.54–5.40) c,d,e,f	3.39 (2.46–5.99) d,e,f	3.16 (2.42–4.83) a,e,f	3.04 (2.45–5.34) a,b,e,f	2.72 (1.89–4.19) a,b,c,d,f	2.89 (2.16–4.60) a,b,c,d,e
MF *p* < 0.05	0.477 (0.347–0.574) d,e,f	0.467 (0.345–0.562) d,e,f	0.461 (0.390–0.503) d,e,f	0.430 (0.361–0.519) a,b,c,e	0.405 (0.366–0.446) a,b,c,d	0.414 (0.379–0.464) a,b,c
MU *p* < 0.05	489 (399–652) d,e,f	501 (407–662) d,e,f	501 (453–568) d,e,f	541 (456–647) a,b,c,e	571 (497–622) a,b,c,d	560 (478–603) a,b,c

Average leaf pair opening (ALPO), Modulation Factor (MF) and total number of Monitor Units (MU). Significant differences (*p*-value < 0.05) between the planning methods are indicated with the letters

**Table 4:** Percentage of points passing the 3%/3 mm gamma criteria

Patient	Plan	Pass rate (%) Global	Pass rate (%) Local
1	Clinical	99.7	95.1
	RP_TO	99.0	93.5
2	Clinical	99.7	93.5
	RP_TO	99.4	94.6
	RP_TO^+^	99.4	95.5
3	Clinical	99.3	94.1
	RP_TO^+^	98.9	94.7
	RP_1 + TO	98.8	94.9
4	Clinical	99.6	94.4
	RP_1 + TO	99.2	94.4

Data for the six plans that reported the lowest MF and the clinical plan corresponding

## Discussion

In this study, the combined use of RP and TO was proposed focusing on HNC radiotherapy VMAT planning. Three workflows were followed using RP together with TO during the optimisation process: the use of an in- house RP model built with TO VMAT plans (RP_TO); the use of an in-house RP model, built with clinical VMAT plans, to obtain the starting optimisation objectives parameters and then further TO (RP_1 + TO); and the use of RP_TO to generate the initial objectives parameters and further TO (RP_TO^+^). The dosimetric impact was assessed on the resulting plans for a set of 20 patients. In addition, for each patient, two more plans were created using TO and RP on its own during the optimisation stage (Clin+TO, RP_1). For the five optimisation strategies and the clinical plans, a dosimetric comparison across the resulting plans was carried out. Additionally, investigation of complexity and deliverability of the plans was performed.

Although the use of RP and TO separately presents advantages over the clinical plans [11, 19], it was shown that for this treatment the combined use of both optimisation methods improves the relationship between balancing OAR doses and PTV coverage, i.e. while maintaining comparable clinically acceptable target coverage, the resulting plans reduced the OAR doses. It was demonstrated that the RP models are able to provide a semi-automatic suggestion of a plan that is a good start for TO. The OAR doses of the plans generated by the RP_TO model, built with previously TO optimised plans, reflect its benefit over the RP_1 model. Therefore, this approach suggests being convenient for centres that does not have TO but that can implement shared RP models. When comparing RP_TO and RP_1 + TO plans no significant difference was reported on OAR sparing, but the first one reduced active planner interaction. Furthermore, when RP_TO is used as the initial plan with subsequent use of TO (RP_TO^+^) this led to the largest dose reduction for all OAR. This reveals that for a new patient, the knowledge-based model that was trained with the plans that were already optimised by TO helped to obtain optimisation parameters that are closer to the desired trade-offs, providing the best starting point for TO. The results are in agreement with Wang J et al. [17] who showed for nasopharyngeal treatments that choosing better optimisation parameters for a plan with the aid of automated planning prior to use MCO led to improved plans.

RP_ TO^+^ plans achieved the lowest average OAR doses for all the dose-volume parameters investigated. Most notably, a significant reduction in mean dose of 7.9 Gy and 7.7 Gy for the left and right parotids respectively was accomplished with respect to the clinical plans. Despite the dose improvement for the OAR, the CI results showed that the dose to normal tissue was slightly compromised. Further work with NTO and the inclusion of a structure, considering normal tissue not being recognised as OAR, as part of the trade-offs exploration objectives is of our interest to investigate improvement [19]. On the contrary, OAR doses with RP_TO plans are higher than with RP_TO^+^ but this plans provided an OAR dose improvement respect to clinical plans while maintaining comparable CI and HI to them, being this influenced by the upper PTVLR objectives used in the RP model. The results from both workflows point to the capacity of TO to decide on the trade-offs pursued for this cohort of patients and agree with ongoing HNC trials that have shown decreased toxicity to the surrounding organs as a result of dose de-escalation to nodal PTV. The implementation of RP and TO for the treatment of this cohort of patients could lead to an improved patient quality of life [34, 35].

The results displayed in Table 2 support the value of TO as a tool for attaining further individualised plan improvements. It is shown that the application of TO as the final step in planning (Clin+TO, RP_1 + TO and RP_TO^+^) resulted in a consistent reduced number of plans failing to meet the OAR constraints. The reason three plan parameters failed with RP_TO^+^ as opposed to two with Clin+TO is that the additional failing plan had the largest PTV1 in the set (1069.0 cc) and this lay outside the range of the plans that constitute the RP models used; therefore, following our trade-offs management, PTV1 D_99_ and PTVLR D_99_ were marginally below the constraint (94.9 and 74.7% respectively). This patient was re-planned with different trade-offs exploration management and the constraints of the PTVs were met at the expense of a 2.4Gy increase in the left parotid mean dose. This example points to the fact that the success of a RP model is dependent upon the quality and robustness of the model itself [20]. Similarly, in the use of TO there is an inherent element of user influence.

The time required to create these plans is equally of relevance. The estimated time in our clinics to plan a HNC case, including optimisation and dose calculation until the final plan is produced, ranges between 50 and 300 min depending on the complexity and the experience of the planner. The use of RP and MCO in clinical practice has proven to reduce planning time [10, 36]. Following the proposed workflows, with the combined use of both approaches, the approximate time taken remained always under 80 min, being affected mostly by the speed of the calculations and the number of optimisation objectives selected rather than by the human time needed or the planner experience. Therefore, using both RP and TO at the same time did not increase our human time at the console, and overall is expected to reduce it as less interaction to reach a clinically acceptable solution is needed.

Kyroudi, et al. [37] reported dosimetric differences between the exploration of the alternative plans generated with MCO and the deliverable plans that are created after this, which take into account physical treatment unit restrictions. In this study, small discrepancies were noticed as well but they were followed by a repetition of the trade-off exploration only in a few cases when we were already in the limit of the dose required to meet the PTVs constraints. Furthermore, an evaluation of the dosimetric delivery parameters was performed to warranty plan deliverability. It was found that the complexity of the plans increased when using RP and TO together. This could be expected as the sparing of the OAR has been improved providing higher quality plans [38]. The highest mean MU value and lowest mean MF and ALPO values were observed for the RP_TO plans suggesting that these plans are more complex than the RP_1 + TO and RP_TO^+^. Although RP_TO^+^ showed better relationship between OAR sparing and PTV coverage, it can be observed that the homogeneity of the targets is slightly better when using RP_TO which has proved to have relation with an increase in complexity [39]. Despite the higher complexity of the plans that used RP and TO, the observed values were within the range of the clinical plans. Further, for the extreme values of the three proposed workflows, in most cases the results from the gamma evaluation showed improvement on the agreement of the delivered and planned dose to the OAR, and concluded that the plans are deliverable clinically.

Future work will focus on the implementation of the RP_TO and RP_TO^+^ workflows in clinical practice and analysis of the physicians’ preference on the resulting trade-offs. The use of the proposed optimisation approaches for other treatment sites is of interest and the opportunity to use RP and TO in combination to escalate the dose delivered to the tumour while maintaining or reducing OAR doses.

## Conclusions

The use of RP combined with TO improved OAR sparing on VMAT HNC radiotherapy plans while maintaining clinically acceptable target coverage. The simultaneous use of both approaches reflects in the resulting plans the individual benefit that they provide. The best OAR sparing was obtained when planning starting by generating a plan with a RP model built with plans optimised with TO and then further individualised TO optimisation. For this workflow, the trade-off between a minor deterioration of the nodal PTV, while maintaining clinically acceptable PTVs coverage, allowed the most significant reduction in OAR doses. RP_TO plans, created with the RP model optimised with TO plans, also provided an OAR dose improvement respect to clinical plans while maintaining comparable conformity and target homogeneity. Plans optimised with RP and TO were proven to be deliverable despite an increase in plan modulation.

## Appendix

**Table 5:** Structures and Objectives used for the RapidPlan models. The rest of the OAR (Lenses, Optic Nerves, Orbits, Optic Chiasm, Larynx, Parotids and Trachea) have only a Line (preferring target) objective with volume, dose and priority Generated

	Volume (%)	Dose (cGy)	Priority
PTV 1 – Upper	0	6800	110
PTV 1 – Upper	3	6700	110
PTV 1 – Lower	98	6350	110
PTV 1 – Lower	100	6250	110
PTVLR - Upper	1	6300	110
PTVLR - Upper	5	5700	110
PTVLR - Upper	50	5400	65
PTVLR - Lower	98	5300	110
PTVLR - Lower	100	5200	120
PRV Brainstem Upper	0	4500	150
PRV Brainstem - Line (preferring target)	Generated	Generated	Generated
PRV Spinal cord - Upper	0	4500	150
PRV Spinal cord – Line (preferring target)	Generated	Generated	Generated

**Table 6:** Number of parameter violations occurred in each group of head and neck plans

Structure	Parameter	a - Clinical	b - Clin + TO	c - RP_1	d - RP_1 + TO	e - RP_TO	f - RP_ TO^+^
PTV 1	D_99_ > 90%	0	0	0	2	1	0
	D_95_ > 95%	1	0	1	1	2	1
	D_5%_ < 105%	0	0	0	0	0	0
	D_2%_ < 107%	0	0	0	0	0	0
PTV LR	D_99%_ > 74.8%	0	0	0	0	1	1
	D_95%_ > 78.9%	1	0	0	0	1	0
PRV Brainstem	D_Max_ < 48Gy	0	0	0	0	0	0
PRV Spinal Cord	D_1%_ < 44Gy	0	0	0	0	0	0
	D_Max_ < 48Gy	0	0	0	0	0	0
Left Parotid	D_Mean_ < 24Gy	10	0	8	0	1	0
Right Parotid	D_Mean_ < 24Gy	10	2	11	1	5	1
Total Fails	Parameters	22	2	20	4	11	3
	Plans	13	2	13	3	7	2

**Table 7:** Mean doses received for larynx and trachea by each planning method when PTV did not originate in these sites

Structure	Parameter	a - Clinical	b - Clin + TO	c - RP_1	d - RP_1 + TO	e - RP_TO	f - RP_ TO^+^
Larynx	D_Mean_ < 40Gy *p* < 0.05	42.0 ± 7.2 d,e,f	39.8 ± 8.3	41.7 ± 6.9 d,e	38.4 ± 8.8 a,c	37.1 ± 7.0 a,c	39.6 ± 7.8 a
Trachea	D_Mean_ - N/S *p* < 0.05	34.8 ± 6.8 b,c,e,f	37.4 ± 6.2 a,c,d,e,f	32.3 ± 5.2 a,b,d,e	35.3 ± 5.5 b,c,e,f	30.2 ± 6.7 a,b,c,d,f	32.6 ± 6.0 a,b,d,e

## Abbreviations

ALPO: Average leaf pair opening
CTV: Clinical target volume
DTA: Distance to agreement
DVH: Dose-volume histogram
HI: Homogeneity index
HNC: Head and neck cancer
IMRT: Intensity modulated radiotherapy
MCO: Multi-criteria optimisation
MF: Modulation factor
MU: Monitor units
OAR: Organs at risk
PRV: Planning organ at risk volume
PTV: Planning target volume
PTV1: Main planning target volume
PTVLR: Low risk planning target volume
QA: Quality assurance
RP: RapidPlan
TO: Multi-Criteria Optimisation-based Trade-Off Exploration
TPS: Treatment planning system
VMAT: Volumetric modulated arc therapy
